# Depressive Symptoms Amongst People with Podoconiosis and Lower Limb Lymphoedema of Other Cause in Cameroon: A Cross-Sectional Study

**DOI:** 10.3390/tropicalmed4030102

**Published:** 2019-07-09

**Authors:** Maya Semrau, Gail Davey, Amuam Andrew Beng, Winston Patrick Chounna Ndongmo, Abdel Jelil Njouendou, Samuel Wanji, Kebede Deribe

**Affiliations:** 1 Centre for Global Health Research, Brighton and Sussex Medical School, Brighton BN1 9PS, UK; 2 School of Public Health, College of Health Sciences, Addis Ababa University, P.O. Box 9086, Addis Ababa, Ethiopia; 3 Parasites and Vector Biology research unit (PAVBRU), Department of Microbiology and Parasitology, University of Buea, P.O. Box 63, Buea, Cameroon; 4 Cameroon and Research Foundation for Tropical Diseases and the Environment (REFOTDE), P.O. Box 63, Buea, Cameroon

**Keywords:** neglected tropical diseases, lymphoedema, podoconiosis, mental health, depression, prevalence

## Abstract

Evidence is emerging that shows elevated mental distress and disorder amongst people with several neglected tropical diseases (NTDs). This study aimed to establish the prevalence of depressive symptoms amongst people with podoconiosis and lower limb lymphoedema of other cause in Cameroon. The study was part of a larger research piece that mapped the geographical distribution of podoconiosis in Cameroon. The Patient Health Questionnaire (PHQ-9; mean) was employed to determine the prevalence of depressive symptoms amongst people with lower limb lymphoedema. Linear regression was used to assess the association between socio-demographic characteristics of participants and depressive symptoms. Internal consistency of the PHQ-9 was estimated through Cronbach’s alpha (α = 0.651). The mean PHQ-9 score among people with lower limb lymphoedema was 3.48 (SD ± 3.25). Using a PHQ-9 score of 5 or above as the cut-off score, 32 participants (38.6%) displayed at least mild depressive symptoms. Unemployment was the only factor that was significantly associated with more depressive symptoms overall. This study shows that depressive symptoms are common amongst people with lower limb lymphoedema in Cameroon. The findings provide support for the integration of psychosocial interventions into packages of care for the management of lower limb lymphoedema.

## 1. Introduction

Lower limb lymphoedema refers to swelling of the lower leg and feet, and may be caused by several neglected tropical diseases (NTDs), including podoconiosis, lymphatic filariasis (LF), and leprosy. Whilst the latter two conditions are caused by parasites and *Bacillus*, respectively, podoconiosis is associated with long-term exposure to irritant red clay tropical soils [1] that occur where ancient volcanic deposits have weathered [2] at high altitude and where there is heavy rainfall.

Approximately 20 million people worldwide are thought to be affected by lymphoedema due to the three diseases—15 million due to LF [3], an estimated 4 million due to podoconiosis [4], and 200,000 registered with leprosy [5]. Recent nationwide mapping in Cameroon found the overall prevalence of lower limb lymphoedema to be 0.8% (with rates varying across regions), of which the majority (62.7%) were podoconiosis cases [6]. This amounts to an estimated 41,556 podoconiosis cases in Cameroon [7]. Other countries with a high burden of podoconiosis include Ethiopia, Uganda, Kenya, and Tanzania [8]. 

Lower limb lymphoedema imposes huge burdens on affected individuals, their families and communities, not only in terms of physical disability [9], but also in terms of mental distress [10], depression [11], stigma [9,12,13,14,15], and loss of economic productivity [16,17,18]. Previous studies have found levels of depression to be 20% amongst people with LF in Nigeria using the Patient Health Questionnaire (PHQ-9) as a screening tool and the Depression module of the Composite International Diagnostic Interview (CIDI) as a diagnostic tool [19], and depressive symptoms to be 12.6% amongst people with podoconiosis in Northern Ethiopia as measured by the PHQ-9 [11]. This, together with the stigma that often accompanies lower limb lymphoedema, significantly increases the burden of disease [20], and also acts as a major barrier to accessing and adhering to morbidity management and disability prevention (MMDP) services for lower limb lymphoedema [21,22]. For LF, there is evidence that the burden of the associated psychological and emotional consequences of the condition is double that of the burden of the physical disease measures [20]. 

This paper reports on the prevalence of depressive symptoms amongst people with podoconiosis and lower limb lymphoedema of other cause in Cameroon. 

## 2. Materials and Methods 

### 2.1. Design

This research was part of a larger study by Deribe et al. (2018) [6] that aimed to determine the prevalence and geographical distribution of podoconiosis in Cameroon, to estimate the population at risk, and to establish which areas qualify for control programs. For this, a population-based cross-sectional survey was conducted to identify people with podoconiosis or lower limb lymphoedema of other cause, using a multi-stage cluster sampling design with stratification of the country by environmental risk of podoconiosis (which includes precipitation, altitude, clay and silt fraction of soil, population density, enhanced vegetation index, distance from water body, and slope of land) [6]. 

The results reported in this paper are those of a sub-study within the larger mapping study, looking at the prevalence of depressive symptoms amongst those people identified as having lower limb lymphoedema (either podoconiosis or lymphoedema of other cause) in the larger study.

### 2.2. Setting

The study was conducted within households of participants in 76 villages from 40 health districts across all ten administrative regions in Cameroon.

### 2.3. Sample

A minimum sample size of 3933 from 80 clusters was established for the larger mapping study based on 95% confidence intervals, an assumed design effect of 15, and the ability to detect a prevalence rate of 0.5% with 0.9% precision and 10% non-response rate. Multi-stage sampling of clusters was used, with 40 health districts selected randomly from the total number of 189 districts for the first stage (divided proportionally according to risk of podoconiosis), and then with two villages selected randomly within each of the chosen health districts during the second stage. Within the 76 villages accessed, all participants who were at least 15 years old and had lived in the health district for at least ten years were surveyed, as long as they were sufficiently healthy to be interviewed [6]. 

Based on this sampling procedure, 10,178 individuals from 4603 households were included in the larger mapping study; of those, 83 (0.8%) people were identified as having lower limb lymphoedema and were included in the study reported here. 

### 2.4. Procedure

In the larger mapping study, all 10,178 eligible participants were surveyed and screened for lymphoedema via a physical examination to detect signs of limb lymphoedema and hydrocele (men only for the latter). Podoconiosis versus lymphoedema of other cause was diagnosed using a clinical algorithm, as follows: all 83 participants who were identified as having lower limb lymphoedema were administered a questionnaire in which they were asked about demographic and socio-economic information, their shoe-wearing and foot care practices, other morbidities, and their leg swelling history test results. Clinical history, physical examination, and disease-specific tests were then used to reach the diagnosis of podoconiosis, and to exclude the common differential diagnoses such as LF, systemic disease, and leprosy [6].

In addition, the questionnaire that was administered to the 83 participants who were identified as having lower limb lymphoedema contained the 9-item PHQ-9 as a measure of depressive symptoms. The PHQ-9 has been used and validated as a measure of depression in a wide range of settings [23,24], including in Cameroon [25,26]. The scale involves asking the person how often they have been affected by a set of nine problems over the last two weeks, for example, having little interest or pleasure in doing things, feeling tired or having little energy, having poor appetite or overeating, and having trouble concentrating. The PHQ-9 scores were classified in this study according to categories that have been widely used [11], including in Cameroon [26], as follows: no depression (0–4), mild (5–9), moderate (10–14), moderately severe (15–19), and severe depression (20–27). Interviews were conducted in either French or English; in cases where the participant spoke neither, translators were used to translate the French or English into their local language. Translators held a degree as a minimum qualification, and were briefed on the study rationale and objectives.

All data were collected using BLU smartphones and uploaded to the Cloud-based LINKS server using software developed and supported by the NTD Support Centre [27].

Ethics approval for this study was obtained as part of the larger mapping study from the Cameroon National Ethics Committee (CNEC) and the Brighton and Sussex Medical School Research Governance and Ethics Committee (RGEC). Participants gave their written informed consent to take part in the study.

### 2.5. Analysis

Data analyses were conducted using IBM SPSS 24. Descriptive analyses (frequencies and percentages for categorical variables, and means with standard deviations for continuous variables) were used to present the socio-demographic and disease characteristics of participants, both for people with podoconiosis and those with lower limb lymphoedema of other cause. Chi-square (categorical variables; using Yates’ correction where expected cell counts were below 10) and independent t-tests (continuous variables) were employed to assess whether there were any differences in socio-demographic and disease characteristics between participants who had podoconiosis versus those who had lymphoedema of other cause.

To determine prevalence of depressive symptoms amongst people with podoconiosis and lower limb lymphoedema of other cause, the mean total PHQ-9 score was calculated. An independent *t*-test was used to assess whether there were any differences in PHQ-9 scores between participants with podoconiosis and lower limb lymphoedema of other cause. Cronbach’s alpha was computed as a measure of internal consistency for the PHQ-9 in order to assess whether the PHQ-9 was able to reliably measure depression within this population; a higher Cronbach’s alpha score indicates a higher likelihood that all items in the scale measure the same underlying construct.

A standard multivariate linear regression model was employed to assess the association between the socio-demographic characteristics of participants and depressive symptoms, with PHQ-9 score (depressive symptoms) as the dependent variable, and the following factors as independent variables: age, sex, level of education, and employment status; the independent variables were selected into the model based on the results of previous studies and prior correlational analyses. Dummy variables were created in SPSS for the categorical variables before they were entered into the regression model. 

A significance level of 0.05 was employed for all analyses.

## 3. Results

Table 1 shows the socio-demographic and disease characteristics of those 83 people identified in the larger mapping study as having either podoconiosis (*n* = 52; 62.7%) or lower limb lymphoedema of other cause (*n* = 31; 37.3%) (out of the total 10,178 people sampled). Participants who were classed as having lower limb lymphoedema of other cause included 15 people who had swelling characteristic of LF (i.e., swelling of the descending type) [28], four who had signs and symptoms of other diseases, four where the swelling had started below 3 years of age, three known-leprosy patients, three with hydrocele, one person with loss of sensation, and one where the lymphoedema had developed after a major surgical procedure.

There were no statistically significant differences for any of the socio-demographic or disease characteristics between those who had podoconiosis compared to those who had lymphoedema of other cause. 

In regard to prevalence of depressive symptoms, the mean PHQ-9 score amongst participants was 3.48 (SD = 3.25), ranging between 0 and 15 (out of a possible score of 27). Cronbach’s alpha for the PHQ-9 was 0.651, which is on the low end of a satisfactory score.

Using a PHQ-9 score of 5 or above as the cut-off score, 32 people (38.6%) with lower limb lymphoedema were classified as having at least mild depressive symptoms (see Table 2). Amongst people who scored above the cut-off score of 5, the mean PHQ-9 score was 6.88 (SD = 2.31).

There were no significant differences in levels of depressive symptoms between people with podoconiosis (mean = 3.38, SD = 3.5) and those with lower limb lymphoedema of other cause (mean = 3.65, SD = 2.82) (*p* = 0.73), though the only people who reported either moderate or moderately severe depressive symptoms had podoconiosis (see Table 2). 

In the multivariate standard linear regression model, only unemployment proved to be a significant predictor (B = 1.87, 95% CIs: 0.13–3.62, *p* = 0.36, *R*^2^ = 0.12), with those people who were unemployed having higher PHQ-9 scores on average than those who were not classed as unemployed, i.e., those who were either employed, retired, or students (mean PHQ-9 score unemployed: 4.89, SD = 2.59, compared to a mean of 3.09, SD = 3.32 for people who were not unemployed); none of the other variables were significant predictors.

## 4. Discussion

Depressive symptoms were found to be common amongst people with podoconiosis and lower limb lymphoedema of other cause in Cameroon, with over one-third of participants (38.5%) displaying at least some degree of depressive symptoms, though the large majority of these were classified as having mild depression levels. 

These results are very similar to those from a study in Ethiopia by Bartlett et al. (2016) [11] that employed a more rigorous method of establishing depression amongst people with podoconiosis, by measuring depression at two time points. In this Ethiopian study, 37.3% of participants displayed PHQ-9 scores of 5 or above at the time of the first measurement. Of those, around a third, i.e., 12.6% of the total number of participants (compared to 0.7% of their healthy neighbours), still displayed PHQ-9 scores of 5 or above two weeks later. It is this more conservative figure of 12.6% that the authors used to estimate prevalence of depression [11]. Given that the initial rate of 37.3% is very similar to the findings in the study reported here, it is possible—though untested—that this could also be the case in Cameroon. Depression is usually established based on symptoms being present for at least two weeks, so a repeat assessment of the PHQ-9 two weeks after the first assessment may possibly result in a more accurate prevalence estimate.

The study in Nigeria that used the PHQ-9 as a screening tool and the CIDI as a diagnostic tool for depression in people with LF [19] did not report the number of participants in their study who screened positive for depression using the PHQ-9, so comparisons cannot be made with that study.

There was little variation in depressive symptoms according to socio-demographic or disease characteristics, with unemployment being the only factor that was significantly associated with higher depressive symptoms overall. This is in line with the LF study from Nigeria, which found unemployment to be predictive of depression [19]. However, due to the cross-sectional nature of these studies, they are not able to verify the causal relationship between unemployment and depressive symptoms for people with lymphoedema. Research from the wider mental health field has demonstrated the possible bi-directionality or negative cycle of these variables, i.e., that factors associated with poverty such as unemployment can cause mental ill-health and that the reverse is also the case, and it would be interesting for future research to explore this association further within this particular population [30]. 

There were several limitations to this study, of which one is that the results may have been biased because the PHQ-9 has—to our knowledge—not been validated amongst this study population in Cameroon (even though it has been validated there amongst people living with HIV [26] and with medical students [25], and has been used in the country within research on numerous occasions); its reliability has also not been established in Cameroon. Since internal consistency for the PHQ-9 was reasonable rather than high in this study, further reliability and validity testing of the PHQ-9 in Cameroon would have strengthened the results of this study. A previous study that validated the PHQ-9 in Cameroon for people living with HIV found that the tool had low sensitivity (though high specificity) for picking up major depressive disorder (MDD), defined as a cut-off score of 10 or more; however, their finding of 3% prevalence for MDD is comparable to the results in the study reported here, where 3.6% of participants had a PHQ-9 score of 10 or more [26]. Other studies, such as the one by Bartlett et al. (2016) [11] mentioned above, have also used more rigorous methods in establishing depression. A further limitation is that no control group was included in the study as a comparator for depressive symptoms (since this was beyond the scope of the larger mapping study), such as people without lymphoedema living in the same circumstances. The relatively small sample size is another limitation, which may have decreased the study’s power in finding significant effects, for example, when comparing the sociodemographic and disease characteristics of participants with podoconiosis to those who had lower limb lymphoedema of other cause, or during the regression analysis. 

Despite the limitations, this study contributes to a growing body of evidence which shows that mental distress and disorder is common amongst people with lower limb lymphoedema such as podoconiosis [10,11,19], thereby greatly increasing the burden associated with the conditions [20]. This research thus highlights further the importance of taking a holistic approach, and integrating psychosocial interventions into MMDP care packages for people with lower limb lymphoedema, to treat not only affected people’s physical symptoms and disabilities but to also improve their mental wellbeing. Only then can the burden associated with these conditions be truly addressed.

## Figures and Tables

**Table 1:** Socio-demographic and disease characteristics of people with lower limb lymphoedema in Cameroon.

Characteristics	Total (*n* = 83)	Podoconiosis (*n* = 52)	Lymphoedema of Other Cause (*n* = 31)
Sex			
Male	42 (50.6%)	28 (53.8%)	14 (45.2%)
Female	41 (49.4%)	24 (46.2%)	17 (54.8%)
Age, mean in years	52.6 (SD = 19.6)	49.83 (SD = 17.42)	57.32 (SD = 22.36)
Marital status			
Married	43 (51.8%)	30 (57.7%)	13 (41.9%)
Widowed	18 (21.7%)	8 (15.4%)	10 (32.3%)
Single	18 (21.7%)	11 (21.2%)	7 (22.6%)
Divorced	4 (4.8%)	3 (5.8%)	1 (3.2%)
Religion			
Christian	67 (80.7%)	42 (80.8%)	25 (80.6%)
Muslim	10 (12.0%)	7 (13.5%)	3 (9.7%)
Animist	1 (1.2%)	0	1 (3.2%)
Other	5 (6.0%)	3 (5.8%)	2 (6.5%)
Level of education			
No formal education	38 (45.8%)	25 (48.1%)	13 (41.9%)
Primary school	27 (32.5%)	15 (28.8%)	12 (38.7%)
Secondary school	17 (20.5%)	11 (21.2%)	6 (19.4%)
Tertiary education	1 (1.2%)	1 (1.9%)	0
Literate			
Yes	40 (48.2%)	27 (51.9%)	13 (41.9%)
No	43 (51.8%)	25 (48.1%)	18 (58.1%)
Employment status			
Employed	60 (72.3%)	40 (76.9%)	20 (64.5%)
Not employed	18 (21.7%)	10 (19.2%)	8 (25.8%)
Student	5 (6.0%)	2 (3.8%)	3 (9.7%)
Retired	0	0	0
Occupation			
Farmer	44 (53.0%)	26 (50.0%)	18 (58.1%)
Businessman/woman	8 (9.6%)	8 (15.4%)	0
Housewife	5 (6.0%)	3 (5.8%)	2 (6.5%)
Labourer	3 (3.6%)	2 (3.8%)	1 (3.2%)
Student	5 (6.0%)	21 (3.8%)	3 (9.7%)
Jobless	11 (13.3%)	7 (13.5%)	4 (12.9%)
Other	7 (8.4%)	4 (7.7%)	3 (9.7%)
Mean number of years in current location	42.2 (SD = 21.0)	39.44 (SD = 19.82)	46.71 (SD = 22.34)
Family member with history of leg swelling			
Yes	18 (21.7%)	11 (21.2%)	7 (22.6%)
No	65 (78.3%)	41 (78.8%)	24 (77.4%)
Disease stage (1–5) *, mean	2.60 (SD = 1.14)	2.65 (SD = 1.11)	2.50 (SD = 1.24)
Years since onset of swelling, mean	17.84 (SD = 13.91)	17.58 (SD = 12.53)	18.29 (SD = 16.18)
Co-morbidity present			
Upper limb lymphoedema	9 (10.8%)	4 (7.7%)	5 (16.1%)
Breast lymphoedema	1 (1.2%)	0	1 (3.2%)
Vulva or penis lymphoedema	2 (2.4%)	0	2 (6.5%)
Hydrocele	3 (3.6%)	0	3 (9.7%)
Chyluria (milky urine)	5 (6.0%)	3 (5.8%)	2 (6.5%)

* The staging system by Tekola et al. (2008) [29] was used, where 1 denotes the least severe disease stage and 5 marks the most severe disease stage.

**Table 2:** Depressive symptoms reported by participants.

Level of Depressive Symptoms (Based on PHQ-9 Scores)	Total (*n* = 83)	Podoconiosis (*n* = 52)	Lymphoedema of Other Cause (*n* = 31)
No depression (0–4)	51 (61.4%)	33 (63.5%)	18 (58.1%)
Mild (5–9)	29 (34.9%)	16 (30.8%)	13 (41.9%)
Moderate (10–14)	2 (2.4%)	2 (3.8%)	0
Moderately severe (15–19)	1 (1.2%)	1 (1.9%)	0
Severe (20–27)	0	0	0
